# Update Your Knowledge of Abdominal MRI: Recent Results, Which May Change Your Practice

**DOI:** 10.2463/mrms.e.2016-0123

**Published:** 2017-03-09

**Authors:** Utaroh Motosugi

**Affiliations:** Department of Radiology, University of Yamanashi, 1110 Shimokato, Chuo, Yamanashi, Yamanashi 409-3898, Japan

**Keywords:** abdomen, magnetic resonance, liver

Magnetic resonance imaging (MRI) plays an important role for the healthcare of patients suffering hepatobiliary and pancreatic disorders. In the era of evidence-based medicine, all radiologists should have the updated knowledge. It is difficult for diagnostic radiologists to check all publications about abdominal MRI, because of busy day-to-day works and of floods of newly published articles. To help keep knowledge updated, the editorial board of *Magnetic Resonance in Medical Sciences* allowed me to pick up several articles, which might change your clinical practice of abdominal MRI, from the literatures published in MRMS and related journals.

PROPELLER or BLADE outstands conventional fast spin echo-T_2_-weighted image (FSE-T_2_WI).^1^ T_2_WI is still key sequence for the assessment of any kind of disease in the abdomen. The conventional FSE sequence has been used for a long time to acquire T_2_-weighted images. However, it will be replaced by a new sequence in which rotating parallel lines in k-space are acquired instead of just parallel lines; PROPELLER (GE Healthcare, Waukesha, WI) or BLADE (Siemens, Erlangen, Germany). Rotating frame-sampling method is more robust for the patients motion including respiration, which offers uniformly high image quality^2^ due to the repeated acquisition of the central part of k-space. In my experience, the high robustness for motion and high image quality of these new type T_2_WI are more than make up for increased acquisition time compared with conventional FSE-T_2_WI.

Diffusion-weighted imaging (DWI) without respiratory triggering shows even better image quality than respiratory triggered DWI, if the same acquisition time is allotted. DWI is also one of the important sequences for detecting small lesions, e.g. small liver metastases, and depicting area of inflammation. The respiratory triggering technique is typically used in abdominal DWI to compensate respiratory motion. However, respiratory triggering prolongs acquisition time. Multiple signal averaging without respiratory triggering is another way to acquire abdominal DWI, which was originally reported as diffusion-weighted whole-body imaging with back-ground body signal suppression (DWIBS). You might think that free breathing acquisition should have more artifact than respiratory triggering acquisition. It could be true if other parameters are all the same, which suggest shorter acquisition time in DWI without respiratory triggering compared with that with respiratory triggering. However, according to the recent results, the image quality can be even better in free breathing method if the same acquisition time is allotted for multiple signal averaging,^3^ i.e. fair comparison in terms of time. You may want to try free breathing DWI instead of respiratory-triggered DWI for simpler and robust acquisition.

Decreased apparent diffusion coefficient (ADC) value of the bone may indicate osteoporosis. Another advantage of DWI is quantifying diffusivity of protons. Interestingly, ADC values can be an indicator of bone mineral density in the thoracic/lumbar vertebra, i.e. osteoporosis.^4^ Primary sclerosing cholangitis (PSC) is one of the risks of osteoporosis as well as a major indication of abdominal MRI. According to the recent preliminary study, osteoporosis due to PSC can be diagnosed by routine abdominal DWI. I am expecting more results about the utility of DWI for bone disorders.

The oral contrast medium is often used for suppressing signals in stomach/duodenum to improve the quality of magnetic resonance (MR) cholangiopancreatography (MRCP). Interestingly, the oral contrast medium can go into the bile duct and unexpectedly decrease the signal intensity of the bile duct. Oral contrast medium (T_2_ and T_1_ shortening properties) is widely used to improve the visibility of biliary/pancreatic duct on two dimensional (2D) MRCP. Even though three dimensional (3D) acquisition is available, 2D MRCP is still used because of its’ very short acquisition time. Despite it is administered to improve visibility of bile duct, it can obscure the cholangiogram due to regurgitation into the bile duct.^5^ The risks of regurgitation include juxtapapillary diverticula, pneumobilia, and history of papillary intervention.^5^ According to these results, MRCP should be also scanned before the administration of oral contrast medium, if the patients have these risks.

Gadoxetic acid-enhanced hepatobiliary phase (HBP) image can be useful to determine stereotactic body radiotherapy (SBRT)-related liver damage. Gadoxetic acid is a hepatobiliary MR contrast agent, which is commonly used for liver imaging. It is well known that HBP image can be used for liver function estimation, since uptake of gadoxetic acid into the liver parenchyma is closely associated with liver function. SBRT has been widely used for the treatment of hepatocellular carcinomas (HCCs) recently. The major complication of SBRT is of cause a damage in the surrounding (non-tumorous) liver parenchyma. Decreased uptake of gadoxetic acid in larger area is supposed to be associated with the risk of increasing Child-Pugh score after SBRT.^6^ Since liver dysfunction due to SBRT is limited in the irradiated area, it might be useful to use gadoxetic acid-enhanced hepatobiliary phase images to describe the risk of patients after irradiation.

The utility of MR elastography for staging liver fibrosis is validated in patients with viral hepatitis.^7^ MR elastography is an emerging technique that enables to measure the stiffness of the organ. The liver cirrhosis/fibrosis staging is one of the best applications of this new technique. Many studies showed that the stiffness measured by MR elastography is well correlated with pathological fibrosis study. According to the results of a systematic review, the sensitivity and specificity of MR elastography for staging cirrhosis are 91% and 81%, respectively.^8^ Besides the cost for implementation, MR elastography is ready for joining the list of routine sequences of liver MR protocol.

Here, I summarized the recent updates about abdominal MRI, which might change your daily practice. It is always important to keep the knowledge updated for offering the best quality of the MR examination to the patients suffering abdominal disorders.
